# 

**Published:** 1877-07-20

**Authors:** 


					﻿VOL. VII.	JULY 20, 1877	No. 7.
THE
SOUTHERN MEDICAL RECORD.
A MONTHLY JOURNAL OF PRACTICAL MEDICINE.
•* QU1CQUID PH2EC1 PIES ESTO KREYIS.”
W
w
w
Ph
ffl
■ V
Hl
•<
o
J—I
fi
w
&
: o
fx
o
E-
l-H
w
H
o
•“i
QC
E
r'
C
GO
<1
w
E
H
H
GO
w
«
Ph
w
co
Ch
00
W
o
w
o
o
K
a
i—i
a
>
I—I
o
00
>
b
b
w
Q
H
Hl
Q
b
Hl
tel
to*
Hi
00
00
o
f
HI
Q
HI
tel
0
JAS. P. HARRISON & CO., Printers.
ATLANTA, GEORGIA.	«	-	.
TERMS: $2.00 per Annum in Advance—Club Rates, 5 Copies for $8.00.
				

